# Supplementary material for: Renal Cell Carcinoma Surgical Treatment Disparities in American Indian/Alaska Natives and Hispanic Americans in Arizona
Source: Int J Environ Res Public Health. 2022 Jan 21;19(3):1185. doi: 10.3390/ijerph19031185 (PMC8834853; doi:10.3390/ijerph19031185)
Supplement: Supplementary file 1 [file ijerph-19-01185-s001.zip › ijerph-1498902-supplementary.pdf]

Supplementary Materials

Supplementary Table S1 Stage I RCC patient characteristics among Mexican Americans in Arizona Cancer Registry.

	Mexican (n=285)	P	US-Born Mexican (n=120)	Mexico-Born Mexican (n=97)	P
Age, median (IQR)	63 (52-71))	0.01	65.5 (57-74))	62 (52-68)	0.01
Gender, n (%)		0.28			0.21
Male	167 (58.6)		77 (64.2)	54 (55.7)	
Female	118 (41.4)		43 (35.8)	43 (44.3)	
Grade, n (%)		0.81			0.38
1&2	163 (74.1)		68 (75.6)	51 (68.9)	
3&4	57 (25.9)		22 (24.4)	23 (31.1)	
Marital Status		<0.001			0.23
Married	148 (51.9)		55 (45.8)	56 (57.7)	
Single	53 (18.6)		21 (17.5)	15 (15.5)	
Separated/Divorced/Widowed	77 (27.0)		39 (32.5)	25 (25.8)	
Unknown	7 (2.5)		5 (4.2)	1 (1.0)	
RCC Histologic Subtype, n (%)		<0.001			0.37
Clear Cell	173 (86.1)		70 (82.4)	64 (90.1)	
Papillary	13 (6.5)		8 (9.4)	2 (2.8)	
Chromophobe	7 (3.5)		2 (2.4)	2 (2.8)	
Others	8 (4.0)		5 (5.9)	3 (4.2)	
Recurrence, n (%)		0.008			0.053
No	177 (79.0)		75 (77.3)	77 (88.5)	
Yes	47 (21.0)		22 (22.7)	10 (11.5)	
RUCC 2013, n (%)		0.06			0.87
1 or 2	218 (76.5)		92 (76.7)	73 (75.3)	
3-7	67 (23.5)		28 (23.3)	24 (24.7)	
High School Education, n (%)		<0.001			0.004
≥90%	34 (11.9)		18 (15.0)	5 (5.2)	
≥70%, <90%	114 (40.0)		57 (47.5)	36 (37.1)	
<70%	137 (48.1)		45 (37.5)	56 (57.7)	
Unemployment, n (%)		<0.001			0.048
<5%	87 (30.5)		42 (35.0)	20 (20.6)	
≥5, <10%	139 (48.8)		60 (50.0)	55 (56.7)	
≥10%	59 (20.7)		18 (15.0)	22 (22.7)	
Poverty rate, n (%)		<0.001			0.045
<25%	66 (23.2)		35 (29.2)	15 (15.5)	
≥25, <50%	96 (33.7)		40 (33.3)	34 (35.1)	
≥50%	123 (43.2)		45 (37.5)	48 (49.5)	

P^1 comparing NHWs and Mexican Americans

P^2 comparing U.S.-born and Mexico-born Mexican Americans

Supplementary Table S2 Associations between race/ethnicity and recurrence in ACR in Stage I RCC patients

	Model 1		Model 2		Model 3	
	**OR (95% C.I.)**	***P***	**OR (95% C.I.)**	***P***	**OR (95% C.I.)**	***P***
**Racial/ethnic minority groups vs. non-Hispanic Whites**						
Non-Hispanic Whites	Reference		Reference		Reference	
American Indians/Alaska Natives	1.09 (0.55-2.17)	0.80	0.86 (0.33-2.23)	0.76	0.93 (0.35-2.53)	0.96
Hispanic Americans	1.04 (0.70-1.57)	0.84	0.90 (0.53-1.52)	0.90	1.02 (0.58-1.78)	0.96
**Mexican Americans vs. non-Hispanic Whites**						
Non-Hispanic Whites	Reference		Reference		Reference	
Mexican Americans	**1.88 (1.07-3.30)**	**0.03**	1.73 (0.85-3.51)	0.10	2.01 (0.95-4.25)	0.07
**U.S./Mexico-born Mexican Americans vs. non-Hispanic Whites**						
Non-Hispanic Whites	Reference		Reference		Reference	
U.S.-Born Mexican Americans	1.84 (0.81-4.20)	0.15	1.22 (0.36-4.14)	0.76	1.29 (0.37-4.53)	0.69
Mexico-Born Mexican Americans	0.52 (0.12-2.17)	0.37	0.37 (0.05-2.75)	0.33	0.40 (0.05-3.03)	0.37

Regression models include age category, gender, marital status, RCC subtypes (exclude NOS), grade, and diagnosis year; Model 2 additionally adjust for nephrectomy type; Model 3 additionally adjust for % High School graduation and % Poverty.

Supplementary Table S3 Impact of nephrectomy type on association between race/ethnicity and recurrence in Stage I RCC patients

	Radical		Partial		
	**OR (95% C.I.)**	***P***	**OR (95% C.I.)**	***P***	$P_{\text{Interaction}}$
**Racial/ethnic minority groups vs. non-Hispanic Whites**					0.22
Non-Hispanic Whites	Reference		Reference		
American Indians/Alaska Natives	0.51 (0.06-4.25)	0.51	1.18 (0.37-1.64)	0.83	
Hispanic Americans	1.50 (0.65-3.46)	0.34	0.74 (0.33-1.64)	0.46	
**Mexican Americans vs. non-Hispanic Whites**					0.28
Non-Hispanic Whites	Reference		Reference		
Mexican Americans	3.12 (1.00-9.76)	0.051	1.31 (0.46-3.71)	0.61	

Regression models include age category, gender, marital status, RCC subtypes (exclude NOS), grade, diagnosis year, nephrectomy type, % high school graduation and % Poverty.

Supplementary Table S4 Sub-distribution hazard regression analysis for RCC specific mortality in ACR

	Adjusted model 1		Adjusted model 2		Adjusted model 3		Adjusted model 4	
	**HR (95%CI)**	**P**	**HR (95%CI)**	**P**	**HR (95%CI)**	**P**	**HR (95%CI)**	**P**
**Surgical Treatment**								
Local Ablation or Nephrectomy					Reference			
No Treatment					**3.85 (1.74-8.52)**	**<0.001**		
**Nephrectomy Type**								
Partial Nephrectomy							Reference	
Radical Nephrectomy							1.49 (0.89-2.49)	0.13
**Race/ethnicity**								
Non-Hispanic Whites	Reference		Reference		Reference		Reference	
American Indians/Alaska Native	0.77 (0.29-2.05)	0.60	0.63 (0.22-1.84)	0.40	0.56 (0.19-1.64)	0.29	0.23 (0.03-2.67)	0.14
Hispanic Americans	1.30 (0.77-2.20)	0.33	1.15 (0.66-2.01)	0.62	1.13 (0.66-1.96)	0.65	1.18 (0.63-2.21)	0.61
**NHWs vs. Hispanic Americans**								
Non-Hispanic Whites	Reference		Reference		Reference		Reference	
Mexican Americans	**3.07 (1.80-5.26)**	**<0.001**	**2.84 (1.57-5.15)**	**<0.001**	**2.62 (1.46-4.69)**	**<0.01**	**3.05 (1.55-6.00)**	**<0.01**
Non-Hispanic White	Reference		Reference		Reference			
U.S.-Born Mexican Americans	**3.97 (2.21-71.3)**	**<0.001**	**3.95 (2.06-7.60)**	**<0.001**	**3.47 (1.80-6.70)**	**<0.001**	**4.02 (1.88-8.62)**	**<0.001**
Foreign-Born Mexican Americans	0.53 (0.07, 3.98)	0.53	0.52 (0.07,3.87)	0.52	0.52 (0.07-3.88)	0.52	0.71 (0.09-5.51)	0.74

Model 1: Adjusted for age category, gender, marital status, RCC subtypes (excluding NOS), grade (1 and 2 vs. 3 and 4), diagnosis year (categorical)
Model 2: Adjusted for age category, gender, marital status, RCC subtypes (excluding NOS), grade (1 and 2 vs. 3 and 4), diagnosis year (categorical), % High School graduation, and % Poverty
Model 3: Model 2 + no surgical treatment vs. treatment
Model 4: Model 2 + radical vs. partial nephrectomy

Supplementary Table S5 Impact of nephrectomy type on association between race/ethnicity and overall mortality

	**Radical**		**Partial**		
	**HR (95%CI)**	**P**	**HR (95%CI)**	**P**	$P_{Interaction}$
**Race/ethnicity**					0.86
Non-Hispanic Whites	Reference		Reference		
American Indians/Alaska Native	1.29 (0.71-2.34)	0.40	1.72 (0.83-3.56)	0.15	
Hispanic Americans	1.22 (0.83-1.79)	0.31	1.20 (0.74-1.93)	0.46	
**NHWs vs. Hispanic Americans**					0.65
Non-Hispanic Whites	Reference		Reference		
Mexican Americans	**2.41 (1.56-3.72)**	**<0.001**	**2.37 (1.37-4.09)**	**<0.001**	
					0.73
Non-Hispanic White	Reference				
U.S.-Born Mexican Americans	**3.70 (2.25-6.10)**	**<0.001**	**3.89 (2.09-7.25)**	**<0.001**	
Foreign-Born Mexican Americans	1.76 (0.79-3.94)	0.17	1.56 (0.63-3.84)	0.33	

Adjusted for age category, gender, marital status, RCC subtypes (excluding NOS), grade (1 and 2 vs. 3 and 4), diagnosis year (categorical), % High School graduation, and % Poverty
